# Matrine Exerts Hepatotoxic Effects via the ROS-Dependent Mitochondrial Apoptosis Pathway and Inhibition of Nrf2-Mediated Antioxidant Response

**DOI:** 10.1155/2019/1045345

**Published:** 2019-10-14

**Authors:** Longtai You, Chunjing Yang, Yuanyuan Du, Yi Liu, Gongsen Chen, Na Sai, Xiaoxv Dong, Xingbin Yin, Jian Ni

**Affiliations:** ^1^School of Chinese Materia Medica, Beijing University of Chinese Medicine, Beijing 100029, China; ^2^Beijing Research Institute of Chinese Medicine, Beijing University of Chinese Medicine, Beijing 100029, China

## Abstract

Matrine, an alkaloid isolated from *Sophora flavescens*, possesses a wide range of pharmacological properties. However, the use of matrine in clinical practice is limited due to its toxic effects. The present study investigated the roles of mitochondria and reactive oxygen species (ROS) in matrine-induced liver injury. Our results showed that treatment of HL-7702 cells with matrine led to significant and concentration- and time-dependent reductions in their viability, as well as significant and concentration-dependent increases in the number of apoptotic cells and supernatant lactate dehydrogenase (LDH) activity. The treatment led to significant increases in the population of cells in S phase and significant reduction of cell proportion in G0/G1 and G2/M phases. It also significantly and concentration-dependently increased the levels of ROS and malondialdehyde (MDA) but significantly and concentration-dependently reduced superoxide dismutase (SOD) activity, level of reduced glutathione (GSH), and mitochondrial membrane potential (MMP). Matrine treatment significantly and concentration-dependently upregulated the expressions of Bax, p53, p-p53, p21, cyclin E, Fas, cleaved caspase-3, caspase-8, and caspase-9 proteins and downregulated the expressions of Bcl-2, cyclin-dependent kinase 2 (CDK2), and cyclin A. It also significantly promoted the cleavage of poly(ADP-ribose)polymerase (PARP), upregulated Kelch-like ECH-associated protein 1 (Keap1) expression, and downregulated the expressions of cellular total and nuclear Nrf2. Matrine significantly inhibited the expressions of downstream oxidoreductases (Heme oxygenase-1 (HO-1) and NAD(P)H:quinone oxidoreductases 1 (NQO-1)) and enhanced the formation of Keap1/Nrf2 protein complex. These results show that the hepatotoxic effect of matrine is exerted via inhibition of Nrf2 pathway, activation of ROS-mediated mitochondrial apoptosis pathway, and cell cycle arrest at S phase. Pretreatment with N-acetyl cysteine (NAC) partially reversed matrine-induced hepatotoxicity.

## 1. Introduction

**Figure 1:**
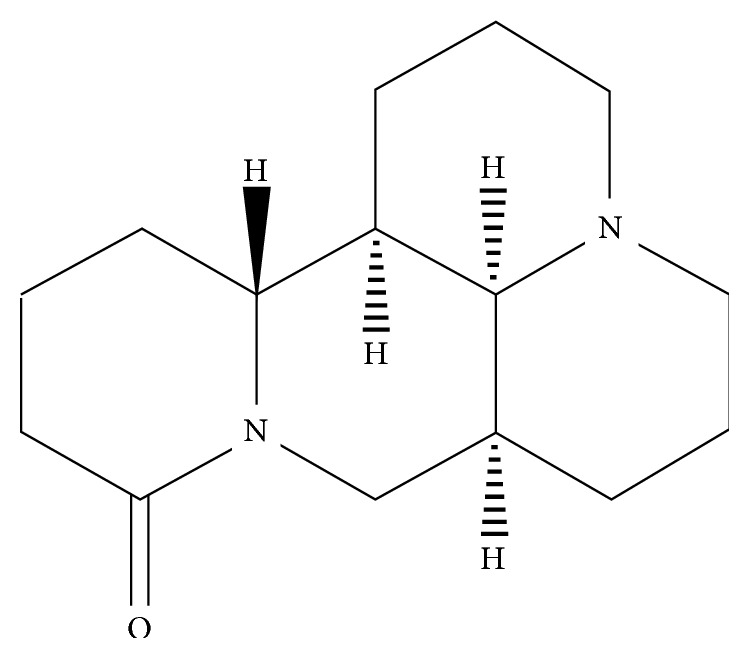
The chemical structure of matrine.

Matrine (dodecahydro3a,7a-diaza-benzo[de]anthracen-8-one) (Figure 1) is an alkaloid isolated from the dry root of *Sophora flavescens*, a plant indigenous to China [1]. *In vitro* studies have shown that matrine possesses a wide range of pharmacological effects, such as anticancer, anti-inflammatory, antibacterial, antiparasitic, antivirus, and antifibrosis properties [2–4]. In Traditional Chinese Medicine (TCM), matrine is used to treat hepatitis, cardiac diseases, skin diseases, and some cancers [5]. It inhibits the proliferation of cancer cells, such as HepG2, Bel7402, HT29, and K562 cells. The involvement of proteins, such as Bax/Bcl-2, Fas/Fas-L, caspase-3, AKT, and JAK2/STAT3, in matrine-induced apoptosis has been reported [6–10]. Reports on the side effects of matrine have increased considerably, and this has limited its use in clinical practice. Studies have shown that matrine exerts hepatotoxic and neurotoxic effects in zebrafish embryos/larvae [11, 12]. In one study, it was reported that treatment with matrine resulted in severe liver damage in mice [13]. However, another study reported that matrine had no significant effect on the viability of HL-7702 cells or body weight of mice, suggesting that it may not be hepatotoxic [14]. Thus, there appears to be confusion about the hepatotoxic effect of matrine, while the underlying mechanism of its toxicity has not been fully elucidated.

Stimulation of cells by xenobiotics or drugs results in the overproduction of ROS and thus oxidative stress. The association of ROS with drug-induced hepatotoxicity is an indication that oxidative stress is one of the major causes of hepatocyte apoptosis and liver dysfunction [15–18]. The activation of nuclear factor-erythroid 2-related factor 2 (Nrf2) has been linked to drug-induced hepatotoxicity [19]. After the translocation of Nrf2 to the nucleus, it interacts with antioxidant response elements (ARE) to modulate intracellular antioxidant responses [20]. Under normal physiological conditions, Nrf2 coexists with Keap1 in the cytosol, and Keap1 directly interacts with Nrf2 to prevent its translocation from the cytosol to the nucleus. High cellular levels of ROS activate the dissociation of Nrf2 from Keap1 and its subsequent transfer to the nucleus. While in the nucleus, Nrf2 binds to ARE and activates the expressions of oxidoreductases such as *γ*-glutamyl cysteine synthetase catalytic subunit (GCLC), HO-1, and NQO-1, resulting in manifestation of its antioxidant effects [21]. Thus, the Nrf2 pathway is considered a major factor regulatory mechanism for reducing oxidative stress.

Apoptosis is a physiological process of autonomous, regulated cell death in response to disease and exogenous stress. It is regulated by two major pathways: receptor-mediated pathway (extrinsic pathway) and mitochondrial-dependent pathway (intrinsic pathway) [22, 23]. Caspases are a family of cysteine proteases which play key roles in cell apoptosis [24]. The extrinsic pathway is initiated by ligation of death receptors and subsequent activation of caspase-8 within a death-inducing signaling complex. On the other hand, the intrinsic pathway is triggered by intracellular stress and subsequent activation by caspase-9. Although both pathways can be activated by various stimuli, both lead to direct activation of downstream effector caspase-3 [25, 26]. The increased production of ROS leads to induction of mitochondrial-dependent apoptosis [27]. Studies have shown that high intracellular levels of ROS result in mitochondrial DNA damage and release of cytochrome *c* from mitochondria to cytosol, thereby triggering caspase-dependent or caspase-independent apoptosis [28, 29].

The HL-7702 cells isolated from a normal human liver exhibit ultrastructural features similar to those of hepatic carcinoma. These cells are useful for assessing drug-induced hepatotoxicity and constitute an ideal in vitro model for cytotoxicity studies [30, 31]. The present study investigated the roles of mitochondria and ROS in matrine-induced liver injury.

## 2. Materials and Methods

### 2.1. Reagents

The HL-7702 cell line was purchased from China Infrastructure of Cell Line Resources. Matrine (batch no. MUST-17030401, purity > 98.72%) was a product of Chengdu Mansite Bio-Technology Co., Ltd. (Chengdu, China). Dulbecco's modified Eagle's medium (DMEM), FBS, trypsin, penicillin, and streptomycin solutions were obtained from Corning (NY, USA), and dimethyl sulfoxide (DMSO), phosphate-buffered saline (PBS), and 3-(4,5-dimethyl thiazol-2-yl-)-2,5-diphenyl tetrazolium bromide (MTT) were products of Solarbio (Beijing, China). Assay kits for SOD, MDA, and GSH were purchased from Nanjing Jiancheng Bioengineering Institute (Nanjing, China). Assay kits for Annexin V-fluorescein isothiocyanate (FITC)/propidium iodide (PI) apoptosis, 4′,6-diamidino-2-phenylindole (DAPI), LDH, MMP, ROS, cell cycle, and bicinchoninic acid (BCA) were purchased from Beyotime (Shanghai, China). Polyclonal antibodies for Fas, Bax, Bcl-2, p53, p-p53 (Ser-15), p21, cyclin A, CDK 2, cytochrome *c*, caspase-3, caspase-9, PARP, cleaved caspase-3, cleaved caspase-9, cleaved PARP, Nrf2, Keap1, HO-1, and NQO1 were products of Cell Signaling Technology (USA). Antibodies for caspase-8 and cleaved caspase-8 were purchased from Abcam (UK). A microplate reader was obtained from Thermo Fisher Scientific Co., Ltd. (USA), and a flow cytometer was the product of BD Biosciences (USA). The ProteoExtract® Cytosol/Mitochondria Fractionation kit was purchased from Millipore (USA), while a protease inhibitor was obtained from Sigma-Aldrich (USA).

### 2.2. Cell Cultures and Treatment

The HL-7702 cells were cultured in DMEM supplemented with 10% FBS and 1% penicillin/streptomycin at 37°C in a humidified atmosphere of 5% CO_2_ and 95% air. After attaining 80-90% confluence, the cells were treated with serum-free medium and graded concentrations of matrine (0–4 mg/mL) for 24 h. Normal cell culture without matrine served as the control group. Cells in the logarithmic growth phase were selected and used in this study.

### 2.3. Cell Viability Assay

The effect of matrine on the viability of HL-7702 cells was assessed using the MTT assay. The cells (5.0 × 10^3^ cells/well) were seeded in 96-well plates and cultured in DMEM for 24 h. Matrine (0-4 mg/mL) was added to the cells, followed by incubation for 72 h. At the end of the third day, 20 *μ*L of 5 g/L MTT solution was added to the wells, followed by incubation for another 4 h. The medium was finally replaced with 150 mL of 0.1% DMSO solution, agitated at 50 oscillations/min for 10 min to completely dissolve the formazan crystals formed, and absorbance of the samples was read in a microplate reader at 570 nm. The degree of cell proliferation was determined at different time points: 24, 48, and 72 h. The assay was performed in triplicate. Cell viability was calculated as follows:

(1) $$\begin{array}{c} \text{Cell viability }\left(\%\right)=\frac{\text{Absorbance of the experimental group}}{\text{Absorbance of the control group}}\times 100. \end{array}$$

### 2.4. Determination of Activity of LDH

The cells were seeded into 96-well plates at a density of 1.5 × 10^4^ cells/well and cultured in DMEM for 24 h. Matrine (0-4 mg/mL) was added to the cells and incubated for 48 h. Then, the cells were trypsinized and the resultant cell suspension centrifuged at 3000 rpm for 10 min to obtain supernatant. The activity of each well was harvested, and LDH activity was determined in the supernatant using the LDH kit according to the instructions on the kit manual.

### 2.5. DAPI Staining

Morphological changes in the nuclei of cells can be visually analyzed by staining DNA with DAPI fluorescent dye [32]. The cells were seeded at a density of 4 × 10^5^ cells/well in 6-well plates and treated with graded concentrations of matrine (0–4 mg/mL) for 48 h prior to staining. Then, the cells were harvested, washed twice with PBS, and fixed with 4% paraformaldehyde for 20 min at room temperature. They were thereafter stained with DAPI solution in the dark for 10 min at room temperature. Changes in the nuclei of stained cells were observed under an inverted Olympus IX71 fluorescence microscope and photographed. The apoptotic cells were identified.

### 2.6. Apoptosis Analysis

The cells were seeded in 6-well plates at a density of 4 × 10^5^ cells/well and cultured for 24 h. Then, matrine at different concentrations (0–4 mg/mL) was added to the medium and incubated for another 48 h at 37°C in the presence or absence of NAC. The cells were thereafter washed with PBS and thoroughly mixed with 295 *μ*L binding buffer. Subsequently, the cells were stained with 5 *μ*L each of Annexin V-fluorescein isothiocyanate and propidium iodide within 25 min at room temperature in the dark. Cell apoptosis was assessed using a flow cytometer fitted with argon laser operated at 485 nm [33].

### 2.7. Measurement of Intracellular ROS

The levels of ROS in HL-7702 cells were determined using the 2,7-dichlorofluorescin diacetate (DCFH-DA) assay. The cells treated with matrine (0–4 mg/mL) were washed with PBS after an initial incubation period of 48 h. Then, 10 *μ*M solution of DCFH-DA was added to the plates and incubated for another 30 min at 37°C. Thereafter, the cells were washed with PBS and injected into the flow cytometer for analysis [34].

### 2.8. Measurement of Lipid Peroxidation

The HL-7702 cells were seeded in 6-well plates at a density of 4 × 10^5^ cells/well and cultured for 24 h. After treatment with graded concentrations of matrine for 48 h, the cells were lysed with ice-cold radio-immunoprecipitation assay (RIPA) buffer and centrifuged at 12,000 rpm for 10 min at 4°C. The level of MDA was determined in the supernatant using the MDA assay kit [35].

### 2.9. Determination of Oxidative Status in the Cells

The HL-7702 cells were seeded in 6-well plates at a density of 4 × 10^5^ cells/well and cultured for 24 h. After treatment with graded concentrations of matrine for 48 h, the activity of SOD and level of GSH were determined using their respective assay kits [36, 37].

### 2.10. Determination of Effect of Matrine on MMP

Following 24 h culturing of the HL-7702 cells seeded in 6-well plates at a density of 4 × 10^5^ cells/well, the cells were treated with graded concentrations of matrine for 48 h and stained with 5,5′,6,6′-tetrachloro-1,1′,3,3′-tetraethylbenzimidazolylcarbocyanine iodide (JC-1) working solution (10 *μ*M) for 30 min at 37°C in the dark. Thereafter, they were washed thrice with PBS and subjected to flow cytometric analysis.

### 2.11. Cell Cycle Analysis

The effect of matrine on the distribution of HL-7702 cells among different phases of the cell cycle was determined using a flow cytometer [38]. The treatment of the cells is referred to under Section 2.9. The cells were then washed with PBS and fixed with 70% ethyl alcohol at 4°C overnight. Tris hydrochloride buffer (pH 7.5) containing 1% RNase A was then added to the plates for 30 min at 37°C in the dark. The cells were subsequently stained with propidium iodide and injected into the flow cytometer for analysis.

### 2.12. Cell Fractionation

The HL-7702 cells (4 × 10^5^ cells/well) were seeded in 6-well plates and cultured for 24 h. After treatment with graded concentrations of matrine for 48 h, the cells were lysed with ice-cold radio-immunoprecipitation assay (RIPA) buffer for 30 min and centrifuged at 12,000 rpm for 10 min at 4°C. Protein concentration in the cell lysate was determined using the BCA assay kit. The mitochondrial and cytosolic fractions of the lysed cells were also separated using the ProteoExtract® Cytosol/Mitochondria Fractionation kit.

Isolation of nuclear fraction: after treatment with matrine, the cells were washed twice with ice-cold PBS and resuspended in ice-cold hypotonic buffer containing 5 *μ*L phosphatase inhibitor, 10 *μ*L phenylmethylsulfonyl fluoride (PMSF), and 1 *μ*L dithiothreitol (DTT) for 10 min. The mixture was then centrifuged at 3,000 rpm for 5 min at 4°C, and the supernatant was discarded. The resultant pellet was washed with cold hypotonic buffer for 30 sec and centrifuged again at 5,000 rpm for 5 min at 4°C to obtain pellet. Ice-cold lysis buffer was added to the pellet for 20 min to sustain the suspension which was thereafter centrifuged at 15,000 rpm for 10 min at 4°C. The resultant supernatant (nuclear fraction) was refrigerated at -80°C prior to use.

### 2.13. Western Blot

Equal amounts of protein were resolved on sodium dodecyl sulfate-polyacrylamide gels (SDS-PAGE) and transferred to polyvinylidene fluoride (PVDF) membrane. After blocking with TBST buffer containing 5% skim milk for 1 h, the membranes were incubated with primary antibodies overnight at 4°C, followed by treatment with corresponding secondary antibodies at room temperature for 1 h [39]. The target proteins were visualized and determined by an intensive ECL detection system.

### 2.14. Immunoprecipitation

After treatment with matrine, HL-7702 cells were collected and lysed with modified RIPA buffer containing protease inhibitor cocktail. The homogenates were centrifuged at 14,000 rpm for 15 min at 4°C. The supernatants were collected and protein concentration was determined by the protein assay kit (Beyotime, Shanghai, China). Whole-cell lysates containing 1 mg of proteins were precleared with protein A-Sepharose beads for 1 h and incubated with 2 *μ*g of anti-Keap1 antibody for 3 h. Immunoprecipitated complexes were washed 3-5 times with RIPA buffer and then boiled in SDS sample buffer for 5 min. The supernatant was then electrophoresed by SDS-PAGE for further Nrf2 antibody immunoblotting.

### 2.15. Statistical Analysis

Data are expressed as mean ± S.D., and statistical analysis was performed using SPSS (17.0). Groups were compared using the LSD test. Values of *p* < 0.05 were considered statistically significant.

## 3. Results

### 3.1. Matrine Induces Cytotoxicity in HL-7702 Cells

**Figure 2:**
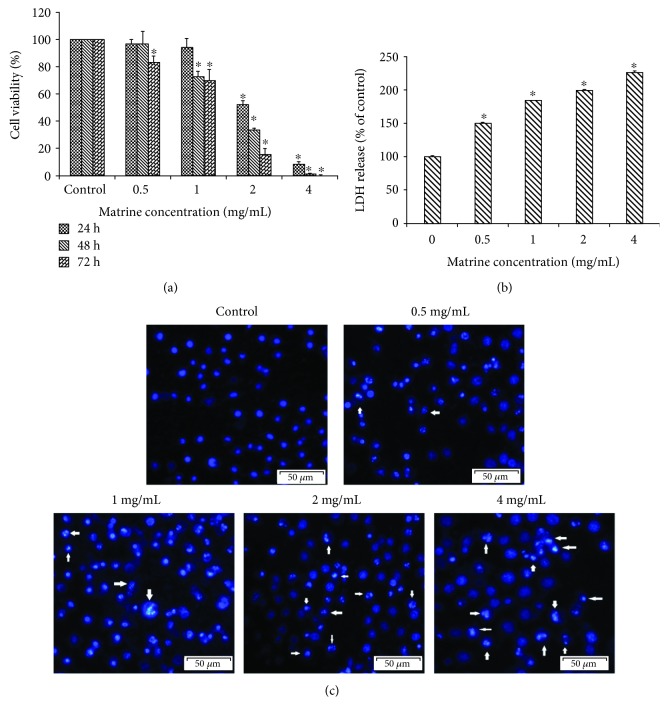
The cell viability and morphology in HL-7702 cells were detected after treatment with various concentrations of matrine. (a) Cell viability was assessed by the MTT assay. (b) HL-7702 cells were treated with matrine at a series of concentrations (0-4 mg/mL) for 48 h. The cell cytotoxicity was evaluated by the LDH assay. (c) The morphology changes of the HL-7702 cell nucleus were examined by DAPI staining and observed by fluorescence microscopy. The arrow markers represent the apoptotic cells. The data are presented as the mean ± S.D. of three independent experiments (^∗^*p* < 0.05 vs. vehicle control).

Compared to the control, the results of the MTT assay showed that matrine obviously inhibited the viability of HL-7702 cells in a dose-dependent and time-dependent manner (Figure 2(a)). The IC_50_ value of matrine for 48 h was 1.446 ± 0.10 mg/mL for HL-7702 cells. When the cell membrane is damaged, LDH is released from the cytoplasm into the extracellular medium, and its release represents disruption of cell membrane integrity. In this study, matrine treatment led to the leakage of LDH observed on HL-7702 cells in a concentration-dependent manner (Figure 2(b)). Next, in order to validate whether the inhibitory effect of matrine on cell growth is related to apoptosis, the morphological changes of the HL-7702 cell incubated with matrine (0-4 mg/mL) were evaluated by staining with DAPI fluorescent dye. As shown in Figure 2(c), treatment with matrine significantly and concentration-dependently promoted apoptosis in HL-7702 cells (*p* < 0.05). Apoptosis was evidenced by chromatin condensation, nuclear fragmentation, and apoptotic body formation.

### 3.2. Matrine Induces Apoptosis in HL-7702 Cells

**Figure 3:**
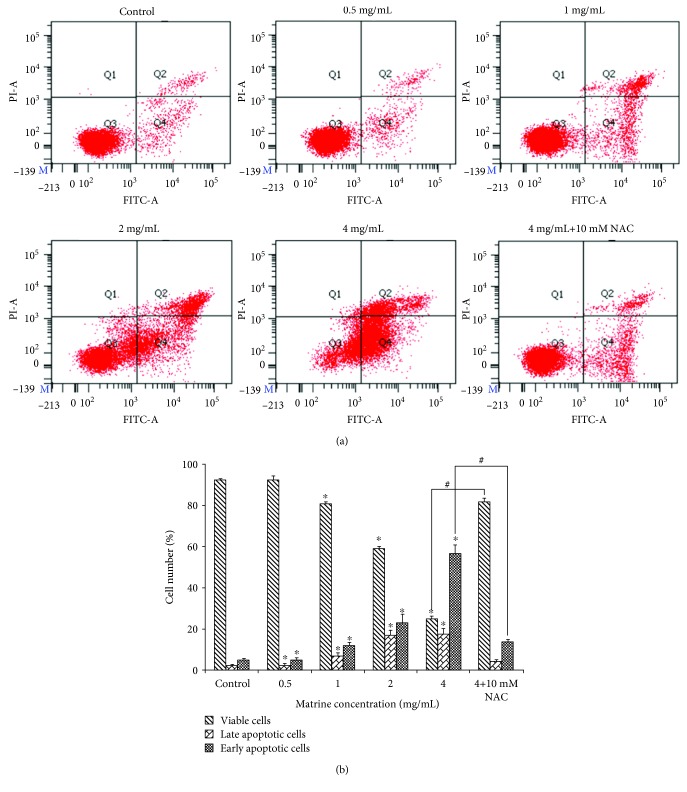
HL-7702 cells were treated with various concentrations of matrine for 48 h. (a) Apoptosis detection with Annexin V-FITC/PI double staining in different groups by flow cytometry. The Q3 region represents viable cells, while the Q4 region represents early apoptotic cells. The Q2 region represents late apoptotic cells, while the Q1 region represents necrotic cells or mechanical damaged cells. (b) Column bar graph of mean cell florescence for viable, early apoptotic, and late apoptotic cells. The data are presented as the mean ± S.D. of three independent experiments (^∗^*p* < 0.05 vs. vehicle control, ^#^*p* < 0.05 vs. matrine 4 mg/mL-treated group).

**Figure 4:**
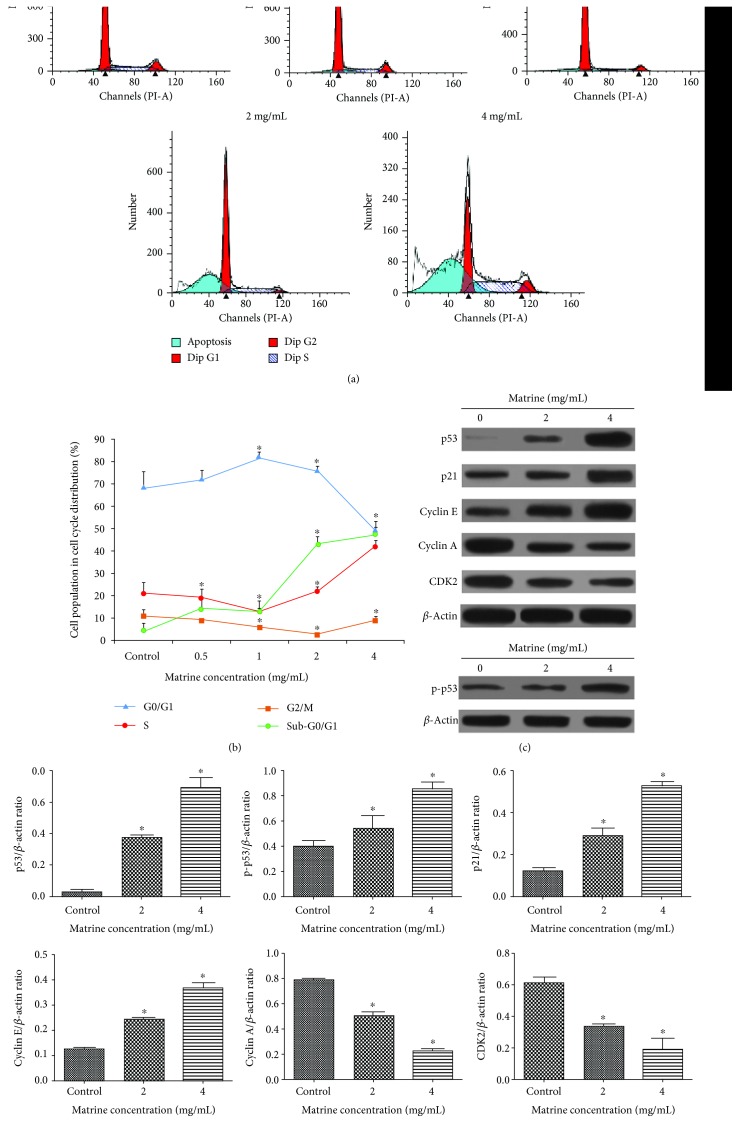
Effect of matrine on the distribution of HL-7702 cells among phases of the cell cycle. (a) Matrine induced cell cycle arrest at the S phases. (b) Each phase of the cell cycle was shown in a polyline diagram. (c) The expression levels of the correlative proteins were measured by western blot. *β*-Actin was used as a loading control. (d) Densitometric analysis was used to quantify these protein-related bands and statistically analyze. The data and each image are expressed as the mean ± S.D. of three independent experiments (^∗^*p* < 0.05 vs. vehicle control).

As shown in Figures 3(a) and 3(b), the percentage of viable cells was significantly lower after incubation with matrine for 48 h. Moreover, the percentage of early and late apoptotic cells was clearly increased in a dose-dependent manner from 5.13% ± 0.70 to 56.80% ± 4.11 and 2.37% ± 0.50 to 17.57% ± 2.89, respectively. Meanwhile, we also detected the presence of a subpopulation of G0/G1 cells corresponding to apoptotic DNA fragmentation by cell cycle analysis (Figures 4(a) and 4(b)). In addition, matrine-induced apoptosis can be partially blocked by the ROS inhibitor NAC (10 mM). These results demonstrate that apoptosis is one of the ways through which matrine induces HL-7702 cell death.

### 3.3. Activation of Oxidative Stress and Mitochondrial Injury by Matrine in HL-7702 Cells

#### 3.3.1. Matrine-Induced Oxidative Stress in HL-7702 Cells

**Figure 5:**
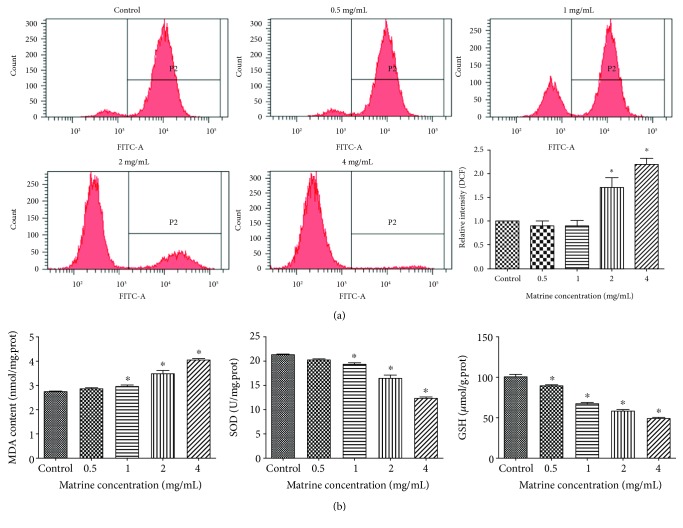
Matrine-induced oxidative stress in HL-7702 cells. Cells were incubated with matrine (0-4 mg/mL) for 48 h. (a) ROS detection with DCFH-DA dye in different groups by flow cytometry. (b) Lipid peroxidation MDA content and antioxidant SOD or GSH level in cells were measured. The data are presented as the mean ± S.D. of three independent experiments (^∗^*p* < 0.05 vs. vehicle control).

The generation of ROS plays an important role in oxidative stress and apoptosis [40]. Compared with the control group, the intracellular ROS and lipid peroxidation MDA level increased in a dose-dependent manner after the cells were exposed to matrine for 48 h (Figures 5(a) and 5(b)). After treatment with different doses of matrine, the antioxidant SOD and GSH levels were markedly reduced (Figure 5(b)). These results indicated that matrine could induce oxidative stress in HL-7702 cells.

#### 3.3.2. Effect of Matrine on Mitochondrial Dysfunction in HL-7702 Cells

**Figure 6:**
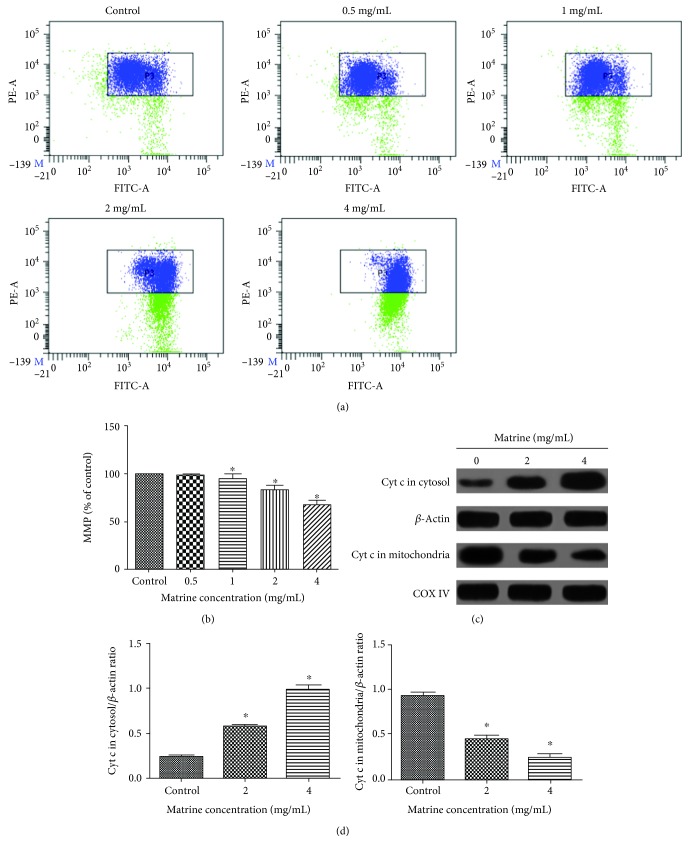
Effect of matrine on MMP in HL-7702 cells. (a) MMP detection with JC-1 staining in different groups by flow cytometry. (b) Column bar graph of mean cell florescent for JC-1. (c) The release of cytochrome *c* in the mitochondria and cytosol was examined by western blotting. *β*-Actin and COX IV were analyzed as the internal control for the cytosolic and mitochondrial fractions, respectively. (d) Densitometric analysis was used to quantify these protein-related bands and statistically analyze. The data and each image are expressed as the mean ± S.D. of three independent experiments (^∗^*p* < 0.05 vs. vehicle control).

**Figure 7:**
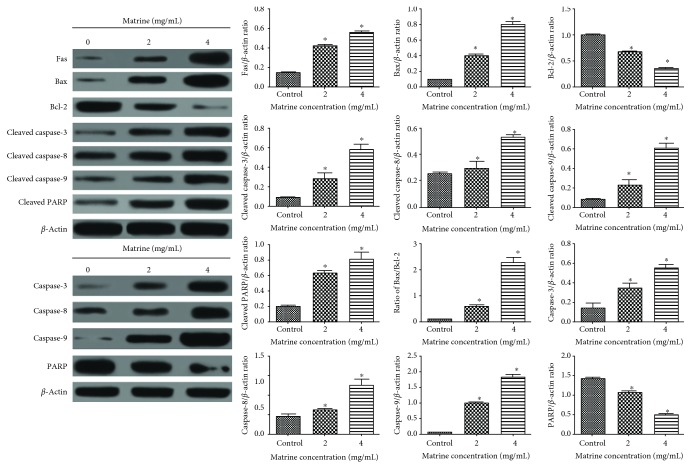
Effect of matrine on the expressions of apoptosis-related proteins as measured using western blotting. Densitometric analysis was used to quantify these protein-related bands and statistically analyze. The data and each image are expressed as the mean ± S.D. of three independent experiments (^∗^p < 0.05 vs. vehicle control).

After confirming the effect of matrine on the apoptosis of HL-7702 cells, we then investigated whether mitochondrial dysfunction mediates matrine-induced apoptosis. Previous studies have shown that the loss of mitochondrial membrane potential (MMP) can increase mitochondrial outer membrane permeability, leading to mitochondrial dysfunction and activation of cytochrome *c* release [41, 42]. Compared with the control group, treatment of HL-7702 cells with matrine induced a significant loss of MMP in a dose-dependent manner (Figures 6(a) and 6(b)). The mitochondria-dependent apoptosis pathways involves signaling of mitochondrial-associated apoptotic proteins, including Bax and Bcl-2. Treatment of HL-7702 cells with matrine increased Bax and decreased Bcl-2 expression in a concentration-dependent manner, which increased the ratio of proapoptotic/antiapoptotic proteins (Figure 7). Moreover, our study demonstrated that matrine significantly increased the release of cytochrome *c* from the mitochondria into the cytoplasm (Figures 6(c) and 6(d)). Taken together, these results indicate that mitochondrial dysfunction is involved in matrine-induced apoptosis of HL-7702 cells.

### 3.4. Matrine Induces Cell Cycle Arrest in S Phase of HL-7702 Cells

To confirm the effects of matrine on cell cycle, the cell cycle distribution of matrine-treated cells was analyzed by flow cytometry. As shown in Figures 4(a) and 4(b), exposure of HL-7702 cells to matrine for 48 h resulted in an obvious increase in S phase cells and a corresponding decrease in G0/G1 and G2/M phase cells when compared with untreated cells. Furthermore, treatment with 2 and 4 mg/mL matrine for 48 h markedly upregulated the expression level of p53, p-p53, and p21 protein in a dose-dependent manner. Cyclin-dependent kinases (CDKs) complexed with corresponding cyclins were involved in cell cycle progression [43]. As shown in Figures 4(c) and 4(d), matrine treatment for 48 h led to significant and concentration-dependent upregulations of the expression of cyclin E and downregulation of CDK2 and cyclin A expressions. Together, these results indicate that matrine induces S phase cell cycle arrest in HL-7702 cells.

### 3.5. Matrine Regulates the Expression of Apoptosis-Related Proteins in HL-7702 Cells

As shown in Figure 7, matrine treatment significantly and concentration-dependently increased the expressions of Bax, cleaved caspase-3, caspase-8, and caspase-9 but significantly reduced the expression of Bcl-2. It also significantly promoted the cleavage of PARP. Furthermore, Figure 7 shows that matrine significantly upregulated the expression of Fas, a typical death receptor. Collectively, these results indicate that activation of death receptors and mitochondrial-dependent apoptotic pathways is involved in matrine-induced apoptosis.

### 3.6. Matrine Inhibits Nrf2 Pathway Activation Associated with Oxidative Stress

**Figure 8:**
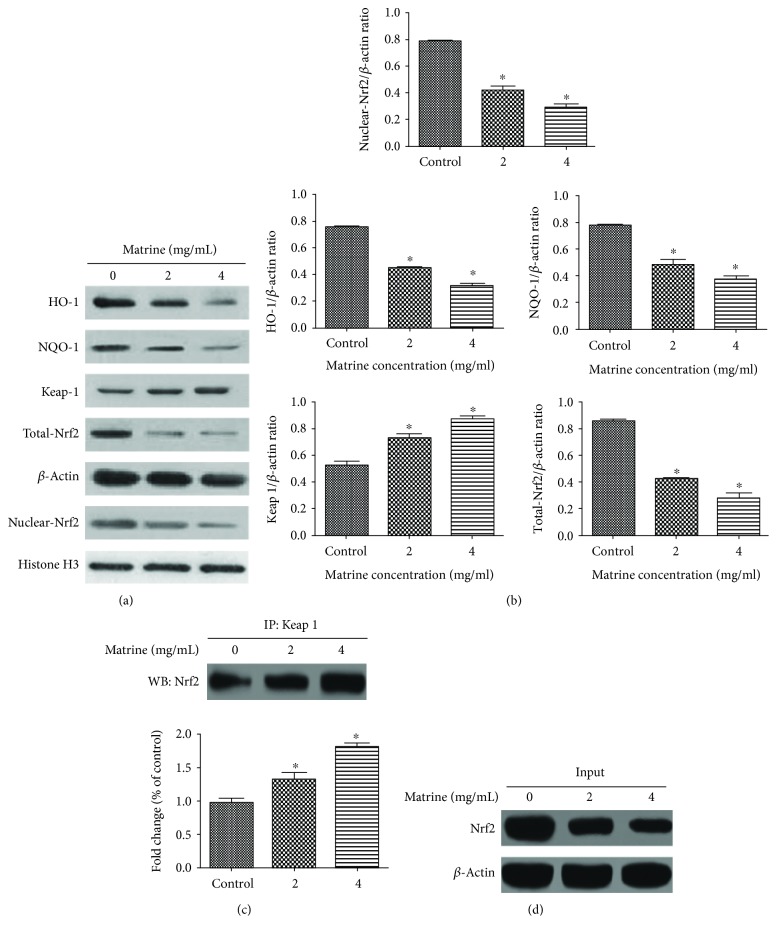
Effects of matrine on Nrf2 pathway. (a) The effects of matrine on the expression levels of Nrf2, Keap1, HO-1, and NQO-1 were measured by western blot after treatment with various concentrations of matrine for 48 h. (b) Densitometric analysis was used to quantify these protein-related bands (Nrf2, Keap1, HO-1, and NQO-1) and statistically analyze. (c) Effect of matrine on the formation of Keap1/Nrf2 complex. (d) Western blot analysis of Nrf2. HL-7702 cells were treated with matrine (0–4 mg/mL) for 48 h. The whole-cell lysates were analyzed by western blot analysis with the indicated antibodies. The data and each image are expressed as the mean ± S.D. of three independent experiments (^∗^*p* < 0.05 vs. vehicle control).

As shown in Figure 8, matrine significantly and concentration-dependently upregulated Keap1 expression and downregulated the expressions of cellular total Nrf2 and the nuclear Nrf2. It also significantly inhibited the expressions of downstream oxidoreductase (HO-1 and NQO-1) expression and promoted the formation of Keap1/Nrf2 protein complex. These results indicate that the oxidative-antioxidant balance of HL-7702 cells is disrupted, leading to inhibition of the Nrf2 pathway, which corresponds to our previous ROS assay results.

## 4. Discussion

Matrine is an alkaloid isolated from *Sophora flavescens*, a plant used in TCM for the treatment of several diseases. It possesses a wide range of pharmacological properties. However, its use in clinical practice is greatly limited due to its speculated toxic effects. Despite the wide speculation about its potential adverse effect, the underlying mechanism has not been fully elucidated.

The results obtained from MTT and LDH assays showed that matrine had a significant cytotoxic effect on HL-7702 cells in a concentration- and time-dependent manner, indicating that it may have hepatotoxicity. Studies have shown that apoptosis is involved in the pathogenesis of hepatocyte injury and liver diseases [44, 45]. Therefore, to assess the association between matrine and apoptosis, the levels of different apoptotic markers in HL-7702 cells after exposure to graded concentrations of matrine were determined. The results showed that matrine induced apoptosis in HL-7702 cells in a concentration-dependent manner.

In a death receptor-mediated apoptotic pathway, the activation of downstream caspase-8 in turn activates Fas ligand (FasL) receptor [46]. Mitochondrial dysfunction which is characterized by increased mitochondrial membrane permeability is usually accompanied by the release of several mitochondrial proteins into the cytosol [47]. Cytochrome *c* is a proapoptotic protein, and its release activates downstream caspase-dependent apoptosis [48]. It has been reported that Bcl-2 prevents the release of cytochrome *c* into the cytosol, and that together with its homologs, it maintains the integrity of mitochondrial membrane [49].

In this study, matrine significantly upregulated the expressions of Fas, cleaved caspase-3, caspase-8, caspase-9, and PARP and the Bax/Bcl-2 ratio. Meanwhile, matrine significantly increased the release of cytochrome *c*. These results suggest that matrine may induce apoptosis in HL-7702 cells via the Fas death receptor-mediated and caspase-dependent mitochondrial apoptotic pathways. Previous studies have shown that matrine activates the mitochondrial apoptotic pathway in esophageal cancer cells [50]. Thus, results obtained in this study suggest that mitochondrial-initiated ROS activation may be also involved in matrine-induced cell death of HL-7702 cells isolated from normal human liver cell and underlie cellular hepatotoxicity.

Oxidative stress plays an important role in the pathogenesis of diseases [51]. The generation of intracellular ROS is closely associated with cell apoptosis [52, 53]. ROS-induced oxidative damage regulates the Bax/Bcl-2 balance, stimulates mitochondrial membrane depolarization, and releases cytochrome *c* from mitochondria into the cytosol [54, 55]. In this study, matrine treatment significantly increased intracellular ROS level, Bax/Bcl-2 ratio, and cytochrome *c* release and significantly reduced MMP. In addition, the level of MDA was significantly increased in matrine-treated HL-7702 cells, an indication that the cells were damaged due to lipid peroxidation. Intracellular SOD and GSH are natural antioxidants that scavenge free radicals such as ROS. The results of this study showed that matrine significantly reduced the antioxidant capacity of HL-7702 cells by decreasing the activity of SOD and reducing GSH levels. In addition, pretreatment with NAC effectively blocked matrine-induced apoptosis. It is likely that matrine induced oxidative damage in HL-7702 cells via the generation of ROS which acted on upstream signaling molecules to trigger cell apoptosis.

It is generally believed that the overproduction of ROS is key in the activation of mitochondria-induced caspase cascade and that the Nrf2 pathway plays an important role in the defense mechanism of cells against oxidative damage [56]. In this study, treatment of HL-7702 cells with matrine significantly downregulated the expression of cellular total Nrf2 and the nuclear Nrf2. However, the formation of Keap1/Nrf2 protein complex was significantly increased by matrine treatment. These results suggest that matrine may have suppressed the induction of antioxidant defenses in HL-7702 cells. It is likely that matrine reduced the antioxidant defense mechanism of HL-7702 cells by targeting the formation of Keap1/Nrf2 complex. It is also possible that matrine may have inhibited nuclear translocation of Nrf2, thereby suppressing downstream antioxidant protein expression. It is likely that matrine-induced oxidative damage may be due partly to the inhibition of its Nrf2-mediated antioxidant activities.

There is an association between oxidative stress and DNA damage [57]. Oxidative damage to DNA due to overproduction of ROS results in rapid activation of p53 and its accumulation within the nucleus [58, 59]. The p21 protein is an inhibitor of cyclin-dependent kinases (CDKs) [60, 61]. Cell cycle is controlled by cyclins, CDKs, and cyclin-dependent kinase inhibitors (CDKIs). Cyclin A is required for both the initiation and elongation of DNA in the late G1 and S phases, while p21 inhibits cyclin A, thereby blocking cell cycle progression from S to G2/M phase [62, 63]. In this study, the expressions of cyclin A and CDK2 were significantly downregulated, while the expressions of p53, p-p53, and p21 proteins were significantly upregulated. These results indicate that matrine may arrest cell cycle in the S phase via activation of p53 expression. It has been reported that p53 triggers apoptosis via the regulation of expression of apoptosis-related proteins such as Bcl-2 and Bax [64]. The results of this study indicate that overproduction of ROS may cause oxidative damage to DNA, which indirectly leads to S phase cell cycle arrest and activation of mitochondrial-dependent apoptosis pathway.

## 5. Conclusions

**Figure 9:**
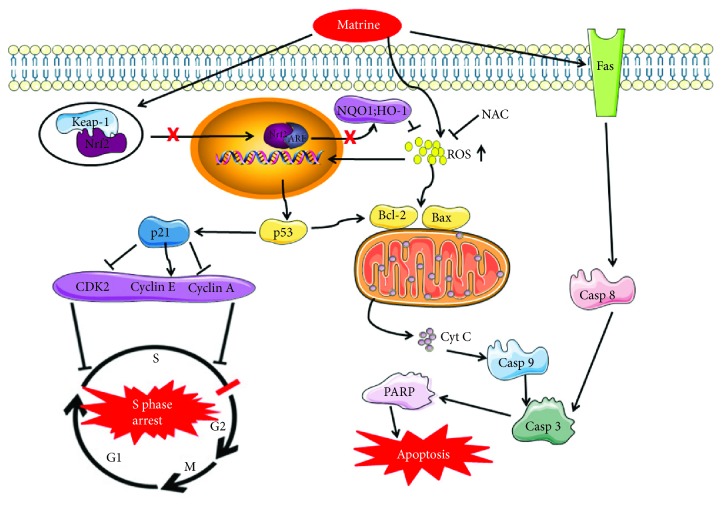
The schematic mechanism of matrine signaling pathway that inhibits cell growth and leads to cell death in HL-7702 cells.

The results obtained in this study show that the hepatotoxic effect of matrine is exerted via inhibition of Nrf2 pathway, activation of ROS-mediated mitochondrial apoptosis pathway, and cell cycle arrest at S phase (Figure 9). Pretreatment with NAC partially reverses matrine-induced hepatotoxicity.

## Data Availability

Parts of data used to support the findings of this study are included within the article. In addition, the data used to support the findings of this study are available from the corresponding author upon request.
